# High Mutability of the Tumor Suppressor Genes *RASSF1* and *RBSP3 (CTDSPL)* in Cancer

**DOI:** 10.1371/journal.pone.0005231

**Published:** 2009-05-29

**Authors:** Vladimir I. Kashuba, Tatiana V. Pavlova, Elvira V. Grigorieva, Alexey Kutsenko, Surya Pavan Yenamandra, Jingfeng Li, Fuli Wang, Alexei I. Protopopov, Veronika I. Zabarovska, Vera Senchenko, Klas Haraldson, Tatiana Eshchenko, Julia Kobliakova, Olga Vorontsova, Igor Kuzmin, Eleonora Braga, Vladimir M. Blinov, Lev L. Kisselev, Yi-Xin Zeng, Ingemar Ernberg, Michael I. Lerman, George Klein, Eugene R. Zabarovsky

**Affiliations:** 1 Department of Microbiology, Tumor and Cell Biology, Karolinska Institute, Stockholm, Sweden; 2 Institute of Molecular Biology and Genetics, Ukrainian Academy of Sciences, Kiev, Ukraine; 3 Engelhardt Institute of Molecular Biology, RAS, Moscow, Russia; 4 Institute of Molecular Biology and Biophysics SD RAMS, Novosibirsk, Russia; 5 Cancer-Causing Genes Section, Laboratory of Immunobiology, National Cancer Institute, Frederick, Maryland, United States of America; 6 Basic Research Program, SAIC-Frederick, Inc., Frederick, Maryland, United States of America; 7 Russian State Genetics Center, Moscow, Russia; 8 State Research Center of Virology and Biotechnology “Vector”, Novosibirsk, Russia; 9 Department of Experimental Research, Cancer Center, Sun Yat-sen University, GuangZhou, People's Republic of China; Karolinska Institutet, Sweden

## Abstract

**Background:**

Many different genetic alterations are observed in cancer cells. Individual cancer genes display point mutations such as base changes, insertions and deletions that initiate and promote cancer growth and spread. Somatic hypermutation is a powerful mechanism for generation of different mutations. It was shown previously that somatic hypermutability of proto-oncogenes can induce development of lymphomas.

**Methodology/Principal Findings:**

We found an exceptionally high incidence of single-base mutations in the tumor suppressor genes *RASSF1* and *RBSP3 (CTDSPL)* both located in 3p21.3 regions, LUCA and AP20 respectively. These regions contain clusters of tumor suppressor genes involved in multiple cancer types such as lung, kidney, breast, cervical, head and neck, nasopharyngeal, prostate and other carcinomas. Altogether in 144 sequenced *RASSF1A* clones (exons 1–2), 129 mutations were detected (mutation frequency, MF = 0.23 per 100 bp) and in 98 clones of exons 3–5 we found 146 mutations (MF = 0.29). In 85 sequenced *RBSP3* clones, 89 mutations were found (MF = 0.10). The mutations were not cytidine-specific, as would be expected from alterations generated by AID/APOBEC family enzymes, and appeared *de novo* during cell proliferation. They diminished the ability of corresponding transgenes to suppress cell and tumor growth implying a loss of function. These high levels of somatic mutations were found both in cancer biopsies and cancer cell lines.

**Conclusions/Significance:**

This is the first report of high frequencies of somatic mutations in *RASSF1* and *RBSP3* in different cancers suggesting it may underlay the mutator phenotype of cancer. Somatic hypermutations in tumor suppressor genes involved in major human malignancies offer a novel insight in cancer development, progression and spread.

## Introduction

We have performed a comprehensive deletion survey of 3p on more than 400 of lung, renal, breast, cervical and ovarian carcinomas (major epithelial cancers) using a defined set of markers, combining conventional LOH with quantitative real-time PCR (QPCR), comparative genomic and NotI microarrays hybridisations [1], [2], [3], [4], [5]. We identified two most frequently affected 3p21.3 regions, LUCA (LUng CAncer) at the centromeric and AP20 at the telomeric border of 3p21.3. Aberrations of either region were detected in more than 90% of the studied tumors suggesting they harbor multiple tumor suppressor genes (TSG) [5], [6], [7].

One of them is *RASSF1* gene (from LUCA region) that can exist in different alternative splicing forms (at least 7 different isoforms). In this work we studied the most important *RASSF1A*, the largest splicing form [8]. Several studies have shown that loss of *RASSF1A* expression occurs because of tumor acquired promoter DNA methylation in many different cancers. For example, *RASSF1A* is silenced by promoter hypermethylation in over 90% of small cell lung carcinomas (SCLC) and clear cell renal cell carcinomas (RCC) and in about 40% of non-small cell lung carcinomas (NSCLC). The gene is able to suppress growth of lung and renal cancer cells in culture and tumor formation in mice [6]. In addition, occasional missense mutations in *RASSF1A* have been reported. *RASSF1A* codes for 340 amino acids. The amino acid sequence of *RASSF1A* contains a predicted diacylglycerol (DAG) binding domain and a Ras association domain. RASSF1A can induce cell-cycle arrest by engaging the Rb-family cell cycle checkpoint [9]. These and other results strongly suggest that RASSF1A is an important human tumor suppressor protein acting at different levels of tumor progression [6].

Another gene is *RBSP3* also called *HYA22* and *CTDSPL*. It exists in two splice forms (A, 265 amino acids and B, 276 amino acids) that map to AP20 region and belongs to a gene family of small C-terminal domain phosphatases that may control the RNA polymerase II transcription machinery [10]. Expression of the gene was greatly decreased in several SCLC and NSCLC cell lines. *RBSP3* showed growth suppression with regulated transgenes in culture and suppression of tumor formation in SCID mice. It was demonstrated that transient expression of both A and B forms resulted in drastic reduction of phosphorylated form of RB protein presumably leading to a block of the cell cycle at the G1/S boundary. After this finding the gene was renamed (RB protein serine phosphatase from chromosome 3). All these features are consistent with classical characteristics of a TSG.

Interestingly, both *RASSF1* and *RBSP3* could collaborate in cell cycle arrest: the former by inhibiting cyclin D1 [9] and the latter by dephosphorylating RB [10]. This supports the hypothesis that TSGs in these two regions could act synergistically [4], [5]. Moreover two other TSGs from these regions could cause increasing mutation frequencies in tumors (*MLH1* from AP20 and *G21/NPRL2* from LUCA) [11], [12], [13].

It is well known that cancer is the result of genetic and epigenetic changes and point mutations is one of the most important mechanisms for the development of cancer [14], [15].

Previously, others and we detected numerous single-base changes/mutations in *RASSF1A* that were believed to be SNPs [8], [16], [17]. Moreover, *RBSP3* mutations were detected in all 14 tumors of different origins expressing the gene [10].

To study the apparently high mutation frequencies of TSG(s) in these regions of 3p21.3, we performed a comprehensive mutation analysis of *RASSF1A* [18], [19] and *RBSP3/HYA22* [10] in several cancers. Here we show that exceptionally frequent single-base mutations occur in these genes in multiple cancer types. The mutations were not cytidine-specific as would be expected if generated by AID [20] or other APOBEC family [21], [22] enzymes. These mutations were not due to RNA editing and appeared *de novo* during cell divisions.

## Results

### Bioinformatics analysis of EST cDNA clones reveals high mutation frequency of *RASSF1* and *RBSP3*

First we examined publicly available EST sequence data for *RASSF1A* and *RBSP3* (for *RASSF1A* Accession No. NM_007182; *RBSP3A*, Accession No. AJ575644, and for *RBSP3B*, Accession No. AJ575645). Sequences with homology below the threshold (see Materials and Methods) i.e. containing multiple distinct mismatches to the annotated genes and unknown nucleotides (N) were not considered. Sequences close to the end of reads were also excluded. The data presented in Table 1 show that the *RASSF1A* and *RBSP3* genes were mutated at extremely high rates. For the *RASSF1A* we considered only 17 clones (mutation frequency per 100 bp, MF = 0.22). Six of them were obtained from cancer tissues and all of them contained mutations (MF = 0.42). Eleven sequences were from normal tissues (four clones with one mutation) and MF = 0.1, i.e. mutation frequencies were statistically significantly different (P = 0.025).

**Table 1:** Bioinformatic analysis of mutation frequency in the *RASSF1* and *RBSP3* genes.

Gene, length*	Type of tissue	Number of ESTs	Total length of ESTs, Kbp	Number of mutations		Mutation frequency, per 100 bp	
				total	nonsynonymous+nonsense+frameshift	total	nonsynonymous+nonsense+frameshift
*RASSF1A*	Cancer+Normal	17	6	13	11	0.22	0.18
	Cancer	6	2.14	9	7	0.42	0.33
	Normal	11	3.9	4	4	0.1	0.1
*RBSP3*	Cancer+Normal	79	35.8	226	137	0.63	0.38
	Cancer	22	10.7	112	77	1.05	0.72
	Normal	57	25.1	114	60	0.45	0.24
insulin	Cancer+Normal	1000	333	0	0	-	-
*P16/INK4α*	Cancer+Normal	20	8.9	0	0	-	-
*GPR14*	Normal	6	7	1	1	0.01	0.01

* Only coding sequences for exons 1 and 2 for RASSF1A and exons 1–8 for RBSP3, the whole ORF for insulin, *P16/INK4α* and *GPR14*.

Eighty one per cent of the *RBSP3* sequences (63 out of 79) contained mutations/mismatches. MF for *RBSP3* ESTs was 0.63. Again it was much higher in clones isolated from cancer (MF = 1.05) than from normal tissues (MF = 0.45). This difference was also significant (P<0.001). The difference was even more pronounced for mutations changing amino acid sequences (MF 0.72 versus 0.24) and similar for RASSF1A clones (MF 0.33 versus 0.1).

The number of available (and mutated) EST sequences was significantly higher for both *RASSF1A* and *RBSP3*, but due to the very stringent criteria many were excluded from analysis.

Importantly, we have also detected hypermutations in other exons of *RASSF1A*, shared with *RASSF1C* (recently shown to be a TSG with a different tissue specificity than *RASSF1A*, see [23]. MF for the exons 3–6 was 0.43 and for the mutations changing amino acids MF = 0.25 and therefore *RASSF1C* is also hypermutated.

A similar bioinformatic analysis was done for the insulin gene (333 bp, complete ORF). No mutations were detected in more than 1000 sequenced clones isolated from cell lines and somatic tissues. In 20 available *p16/INK4a* (exons 1–3, 447 bp) clones sequenced from cancer and normal cells we found no mutations and in 6 clones for *GPR14* (1170 bp) only 1 mutation was found in cancer cells (MF = 0.01). In our experiments described below (see next Section and Section “Different mutations frequencies in other genes”) in 31 sequenced *GPR14* clones no mutations were found indicating that this mutation is rather rare. The mutation frequency for GPR14 was statistically significantly different as compared both to the RASSF1A and RBSP3 (P = 0.01).

### Frequent mutations in *RASSF1A* in human carcinomas, cancer and haematopoietic cell lines

During analysis of *RASSF1A* we have isolated several mutant clones including one double mutant [16]. This high frequency of mutations was surprising since for *RASSF1A* and other candidate genes in the AP20 and LUCA regions the mutation frequencies were reported to be low to none [6], [19]. At the same time many polymorphisms were recorded for *RASSF1A* and in many cases it was not clear whether it was a real single nucleotide polymorphism or somatic mutation in cancer cells because control normal cells were not available [8]. Importantly, in all these studies single-strand conformation polymorphism (SSCP) and direct sequencing from PCR products was used. The admixture of stroma, blood vessels, lymphocytes and other normal cells would hamper detection of mutations using these methods (see M/M). Tumor heterogeneity creates additional problems for recognizing mutations. Therefore we decided to re-investigate the mutational status of *RASSF1A* in multiple tumor types including primary tumors and cancer cell lines. First, *RASSF1A* cDNA was isolated from an RCC biopsy (T356) and the surrounding normally looking kidney parenchyma (N356). Several cDNA clones were sequenced. In six clones derived from normal kidney parenchyma, no mutations were found. However of seven clones from the tumor tissue, mutations were detected in three (P = 0.14). All were A to G substitutions. To exclude RNA editing, genomic DNA from the same patient was isolated and the first two exons (DAG domain) of *RASSF1A* were amplified by PCR. Several clones derived from normal and tumor tissue were then sequenced: all six clones from the tumor biopsy showed mutations while of the fourteen analyzed clones from normal tissue only one was mutated (P<0.001). The observed mutations in the cDNA from tumor cells were not created by RNA editing because the mutations were detected also on genomic DNA level. Normal cell contamination and high expression of *RASSF1A* in normal cells, compared to cancer cells, can explain the different ratios between mutated and normal *RASSF1A* clones from cDNA and genomic DNA. Most surprising was the fact that with the exception of two genomic clones from the tumor biopsy with deletion of C at position 254 (Accession No. NM_007182), all other detected mutations were in different positions.

As a control we amplified *GPR14* from the same patient and sequenced 10 clones from cancer and from the surrounding normal tissue. No mutations were found proving that high mutation rate is specific for the *RASSF1A* gene.

To check whether different mutations in the same tumor occurred due to the tumor heterogeneity or some other mechanism(s), we isolated and sequenced *RASSF1A* clones (only exon 1 and 2; 391 bp) from genomic DNA of four RCC cell lines. In TK164 all three and in KRC/Y (2+2) all four sequenced clones contained mutations. In TK10, among 22 clones, 9 were mutated. Importantly, the majority of clones contained different mutations. Only one clone was sequenced from Caki1, and it was mutated.

We also sequenced this gene from genomic DNA of four lymphoid cell lines (BL2 and RAMOS are Burkitt cell lines, and IARC171 and MutuIII are lymphoblastoid cell lines) and the results were very similar to the RCC cell lines (Table 2). Altogether, among 84 sequenced clones 55 contained mutations that in most cases differed. MF in *RASSF1A* in these experiments was between 0.14 and 0.70.

**Table 2:** Mutations in *RASSF1A* exon 1and 2 in different cell types.

Locus *RASSF1A*/Cell line	Description	Number of clones, mutated+nonmutated	Mutation frequency, per 100 bp	Total number of mutations	Deletions	Transitions over transversions	Mutations of G/C nucleotides
IARC171	Burkitt's lymphoma derived cell line	11+7	0.23	15	no	2	9
BL2	Burkitt's lymphoma derived cell line	10+0	0.70	25	3	1	16
RAMOS	Burkitt's lymphoma derived cell line	11+2	0.56	26	1	1.2	15
mutuIII	Burkitt's lymphoma derived cell line	7+0	0.56	14	1	1.6	9
TK10	renal cell carcinoma derived cell line	9+13	0.15	12	no	0	8
TK164	renal cell carcinoma derived cell line	3+0	0.28	3	no	2	2
KRC/Y	renal cell carcinoma derived cell line	2+2	0.14	2	no	0	no
T356(RCC)	renal cell carcinoma biopsy	6+0	0.51	11	3	0.6	5
N356(RCC)	normal renal cell biopsy	1+13	0.02	1	no	0	no
Caki1	renal cell carcinoma derived cell line	1+0	0.28	1	no	0	1

In all further experiments, we analyzed genomic DNA (exon 1 and 2 for *RASSF1A* and the whole *RBSP3* transgene in pETE vector) if not specially mentioned.

### Mutations in *RASSF1A* can be generated *de novo*

To distinguish between the possibility that different mutated *RASSF1A* genes were mutated at once (“burst of mutations”) or were constantly generated over time, we performed experiments with single cells. In this experiment BL2 cells, (which previously showed the highest rate of mutation: 10 clones with 25 mutations), were diluted and plated into wells with an expected frequency of 0.3 cells per well. Three randomly selected wells (designated as BL2-cl.1, 2 and 3) containing single cells were expanded and further analyzed. DNA was isolated from these clones after 10 days (approximately 10 divisions, 10^3^ cells).

The results were as follows: for BL2-cl.1, five of 10 sequenced clones were mutated (mutation frequency per 100 bp (MF), was 0.14), for BL2-cl.2, five of 13 clones (MF = 0.15; two clones contained T43T mutations with codon changed from ACA to ACG) and for BL2-cl.3, three of 17 clones were mutated (MF = 0.07; two clones contained N70G mutations). Altogether 16 single base pair mutations were detected, all were transitions and only five of them showed mutated G or C. This experiment clearly shows that mutations in the *RASSF1A* locus could be generated *de novo* during cell proliferation.

**Figure 1:**
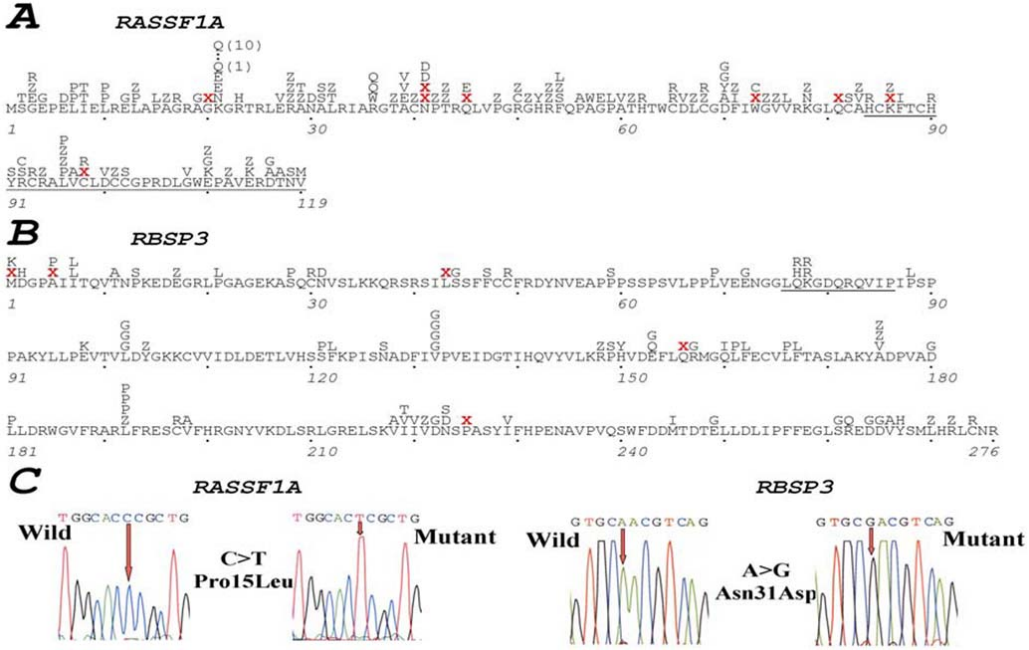
Mutations in *RASSF1A* and *RBSP3* in natural and experimental tumors. Position of mutations detected in *RASSF1A* and *RBSP3* is shown in A and B respectively. Examples of mutations are shown in C. For *RASSF1A* only mutations in coding sequences of exons 1 and 2 are shown. Mutations in the whole coding part of RBSP3 are shown. Red “X” marks stop nonsense mutations or deletions. “Z” designates synonymous mutations.

The complete list of 129 mutations (111 mutations were different) found in exons 1 and 2 of *RASSF1A* in all experiments is shown in Table S1A. See also Table 2 and 3 and Figure 1*A*. Altogether 144 clones were sequenced (56,3 KB) and the average frequency of mutations was 0.23/100 bp for transcribed sequences and 0.17/100 bp for coding sequences. Among them, there were four nucleotide changes that occurred in non-coding 5′, three stop (nonsense) and five frameshift (deletions) mutations. Of the remaining 127 mutations, 82 were missense and 35 synonymous.

**Table 3:** Experimental mutations frequency in the *RASSF1A* and *RBSP3* genes.

Gene, length	Number of sequenced clones	Total length/coding sequences, Kbp	Number of mutations		Mutation frequency, per 100 bp	
			total	In coding region	total	In coding region
*RASSF1*, exons 1–2	144	56.3/51.4	129	89	0.23	0.17
*RASSF1*, exons 3–5	98	50.6	146	145	0.29	0.29
*RBSP3*, exons 1–8	85	85.3/70.6	89	79	0.10	0.11

### *RBSP3* is also hypermutated in various cancers

During previous analyses of small cell lung carcinoma (SCLC) cell line N417, two RCC, one breast carcinoma (BC) and two ovarian carcinoma (OC) biopsies that all expressed *RBSP3*, we detected mutations in the *RBSP3* cDNA in all six cases [10; see Table S2A].

To test whether the hypermutation feature is a characteristic only of the *RASSF1A* gene or a more general phenomenon, we similarly analyzed the recently identified multiple tumor suppressor gene *RBSP3* located in AP20, 3p21.3 telomeric region [10].

Using RT-PCR, cDNA was isolated from two of each RCC, BC and OC biopsies and the SCLC cell line N417. Multiple clones were sequenced. Results, presented in Table S2A, Table 3 and Figure 1B, showed that almost all isolated clones suffered mutations. As reverse transcriptase used in RT-PCR has a significantly higher error rate than other polymerases used in PCR, we attempted to reproduce the observed high mutation rate at the genomic DNA level, as in the case with *RASSF1A*. Unfortunately, it was difficult to perform this experiment on the genomic *RBSP3* due to the large size of the gene (more than 120 kb), numerous small exons (at least 9), and high GC content (reaching 100% in some regions). However this problem was solved using cloned *RBSP3* in SCID mice.

### *RBSP3* revealed high mutability in SCID mice on genomic level

SCLC cell line ACC-LC5 and RCC cell line KRC/Y were transfected with *RBSP3A* and *RBSP3B* splicing isoforms in the pETE vector and stable cell clones were isolated. Four of these clones (AHA1 and AHB1 for ACC-LC5 and KHA4 and KHB9 for KRC/Y) were inoculated into SCID mice (see M/M).

Cell clones KHA4 and KHB9, containing *RBSP3A* or *RBSP3B* were grown *in vitro* in parallel with tumors in SCID mice. After 8 weeks DNA was isolated from grown tumors and cell lines, and the *RBSP3A* and *B* genes were amplified by PCR from pETE vector and cloned. Again multiple clones were sequenced and results of the experiment are shown in Table 4 and Table S2B. Only 30% of *RBSP3* KHA4 and KHB9 plasmid clones were mutated *in vitro*, as compared to 85% mutated clones after growth in SCID mice. This difference according to Fischer test is statistically significant (P<0.001).

**Table 4:** Mutation frequency of the *RBSP3A* and *RBSP3B* genes *in vitro* and *in vivo* in the gene inactivation test.

Gene/cell clone	*In vitro*		*In vivo*	
	tested	mutated	tested	mutated
*RBSP3A*/KHA4	11	3	13	10
*RBSP3B*/KHB9	12	4	15	14
Total	23	7	28	24

In summary, in *RBSP3* experiments we identified 89 mutations among which 79 were individually distinct (see Tables S2A and S2B). The average frequency of mutations was 0.10/100 bp for transcribed sequences. This frequency is more than 0.11/100 bp for coding sequences (see Table 3 and Figure 1B). Among them, seven nucleotide changes occurred in non-coding regions and five were frameshift (deletions) mutations. Of the remaining 77 mutations, 68 were missense and 9 synonymous.

Thus, the mutation frequency was 2.5 fold less than for the first two exons of the *RASSF1A* (see above). The significant difference in mutation frequencies could be accounted for by differences in nucleotide composition of the genes, or it could reflect intrinsic differences in the hypermutation rates of the genes. It could also be important that for the *RBSP3* the whole gene was sequenced while for the *RASSF1A* only its 5′ end.

### *RASSF1A* and *RBSP3* amplified by PCR from *E.coli* DNA don't show high frequency of mutations

We have performed PCR amplification of *E. coli* DNA containing plasmids (i.e. total DNA isolated from *E.coli* containing mixture of genomic and plasmid DNA) with these two genes. For each gene ten and four ng of DNA was used. Unfortunately lower amount of *E. coli* DNA didn't produce sufficient amount of PCR products for further cloning. Ten clones in each experiment were sequenced and no mutations were detected. These results indicate that the observed hypermutation rate of *RASSF1A* and *RBSP3* cannot be explained by PCR polymerase errors.

### Search for founder mutations in *RASSF1A* in single cell clones

**Figure 2:**
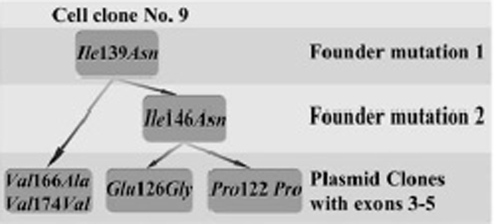
Flow chart showing accumulation of mutations (including two founder mutations) in *RASSF1A* exons 3–5 in the single cell clone #9. Synonymous mutation Pro122Pro was caused by nucleotide change ATC→AAC. Mutation GTC→GTA also didn't result in any amino acid change (Val174Val).

The main idea of this experiment was the following. If a mutation originates in the cell and not in the tube *in vitro* then in the cell population grown from one cell some fraction (depending on the number of alleles present in the single cell) of plasmid clones should contain the same (i.e. a founder) mutation. To perform this experiment we isolated 15 single cell KRC/Y clones as described in the section “Mutations generated de novo”. In this case we grew the cells for three weeks to obtain more DNA and generate more mutated clones. KRC/Y cells were used instead of BL2 cells as it was easier to detect that we have one cell in the well. However, we of course cannot exclude that in some of the 15 selected wells there were more than one cell. *RASSF1A* exons 3–5 were tested in this experiment (see M/M) as they were more easily isolated than exons 1 and 2 and contained more sequence information (516 nt vs. 391 nt). Moreover, according to EST sequence data this part of *RASSF1A* has higher MF. From each PCR reaction 10 plasmid clones were selected and DNA was isolated. However, due to different technical problems (no or rearranged insert, bad quality DNA or sequencing, etc.) usually only six-seven plasmid clones were further analysed. Totally 98 plasmid clones were sequenced (Table 5). One founder mutation was detected in all cell clones and in 46 (47%) of plasmid clones. It was a change of A to G (nt26735, Accession No. AC002481) just at the border of intron 2 and exon 3. This mutation destroyed the splice acceptor site AG/G and thus inactivated the gene. As this founder mutation appeared in all single cell clones most probably it originated before we started to do this experiment. Forty other founder mutations specific for each cell clone were also detected (see Table 5 and Table S1B). Interestingly in one case it was possible to construct a tree showing how founder mutations were accumulated. First it was only one mutation than two and then additional independent mutations (Figure 2).

**Table 5:** Founder mutations in single cell KRC/Y clones.

Cell clone	Mutation	Number of sequenced plasmid clones with founder mutation
All 15 clones	nt26.735(A→G)	46
1	T196T	3
2	R240R, K241R	2
3	D157N	4
4	I139T	5
4	V225A	4
5	N155S	3
8	K232R	3
9	I139N	3
9	I146N	2
10	L256W, P274P	2
12	E126G	2
14	L260S	4
15	D262G	3
Total founder mutations		86

### Different mutation frequencies in other genes

Similar sequencing experiments were performed with insulin and albumin genes isolated from KRC/Y cell line (see M/M). In contrast to *RASSF1A* and *RBSP3* results, only one of 21 sequenced insulin genomic clones (999 bp including complete ORF) and one of 19 albumin cDNA clones (700 bp, exons 12–15) were mutated (MF for both genes was less than 0.01). However in both cases, we could not exclude the possibility of polymorphisms. Additionally, two more genes were tested for mutations in genomic DNA. *GPR14* (G protein-coupled receptor 14, 1018 bp) and transcription elongation factor A (SII) *TCEA1* (1066 bp) were PCR amplified (see M/M) from DNA of KRC/Y cells and 11 clones for each gene were sequenced. All clones contained normal copies of the gene. No mutations were found in other 3p21.3 candidate genes: *BLU* (15 clones were sequenced), *101F6* (6 clones), *PL6* (6 clones) after KRC/Y stable clones containing these genes were inoculated into SCID mice. Moreover, for already mutated mut*FUS1* (10 clones) and mut*P53* (6 clones) no additional mutations were found (data not shown).

### Mutations in *RASSF1A*, *RBSP3A* and *RBSP3B* are not generated by AID or APOBEC related mechanisms

**Figure 3:**
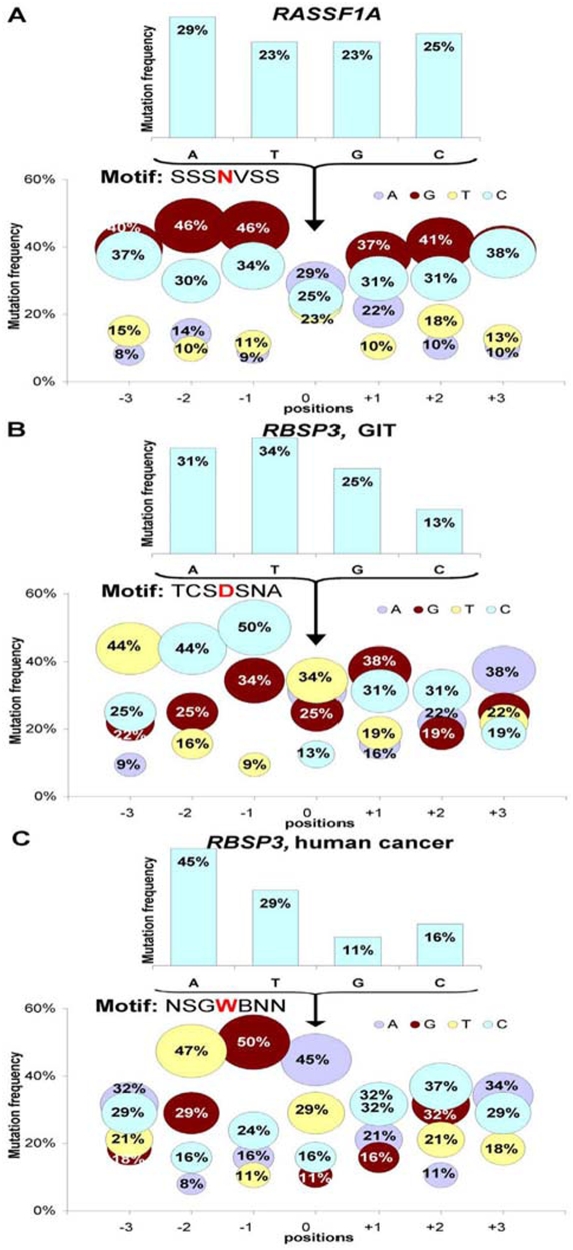
Distribution of mutations in *RASSF1A* (*A*) and *RBSP3* (*B* and *C*). For *RASSF1A* all mutations were analyzed. For *RBSP3* mutations found in GIT (*B*) and in human cancer (*C*) were analyzed separately. Bubble graphs depict the proportion of substitutions occurring at each of the four bases in the *RASSF1A* and *RBSP3*, depending on the distance from the mutated nucleotide (No. 0). N, any nucleotide;B = C, G or T; D = A, G or T; S = G or C; V = A,C, or G; W = A or T.

It has been recently shown that the activation-induced cytidine deaminase (AID) is responsible for somatic hypermutations in activated B cells. Moreover hypermutations generated by this enzyme in oncogenes can cause malignancies in haematopoietic cells [24]. Although much remains to be learned concerning AID, several target sequence motifs for the mutations have been identified, namely WRC, RGYW and DGYW causing C/G mutations. The large family of APOBEC genes, also shown recently to mutate genes on DNA level [21], [22], mostly targeted the RCW motifs causing mutations in C/G. Therefore, we checked whether these motifs were targeted or more frequent in *RASSF1* and *RBSP3* sequences when compared to the stable insulin gene. The frequency of the WRC motif per 100 bp varies from 12.3 to 14.3 for *RASSF1* and *RBSP3* genes, and the insulin gene contains 16.5 such motifs per 100 bp. Other motifs showed the same distribution (also higher in the insulin gene), arguing against the involvement of these enzymes in hypermutating the *RASSF1* and *RBSP3* genes described here. Indeed, the APOBEC and AID enzymes cause mutations almost exclusively in C/G nucleotides, while we observed mutations of all 4 nucleotides (Figure 3). The results actually showed that mutations in A/T were even more frequent than in C/G. We tried to find a recognition motif. We studied all mutations (Figure 3A) or a subset of mutations (Figures 3B and 3C), but no obvious motifs have been yet identified.

More studies are needed to resolve this question as this pattern can be different in normal and cancer cells and could be dependent on nucleotide composition of a gene. These small differences in patterns could mask the recognition motif.

### *RASSF1A* and *RBSP3* mutants from RCC biopsy and lung cancer cell line have significantly reduced growth-inhibiting activity

**Figure 4:**
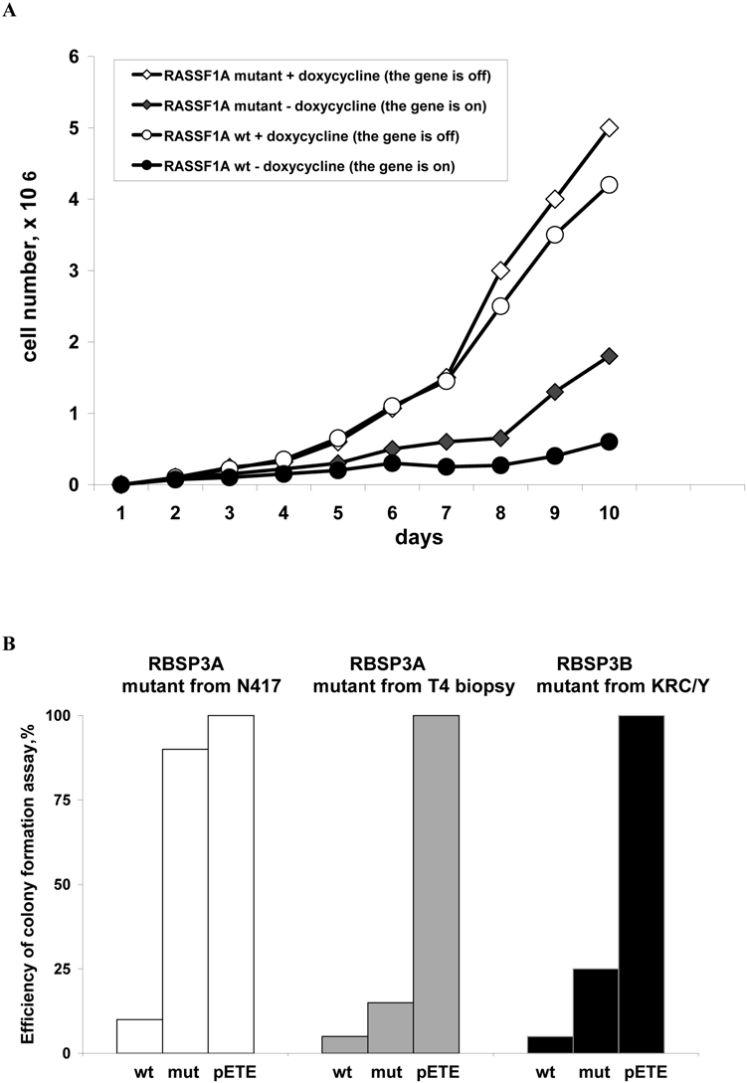
Reduced growth inhibiting activity of *RASSF1A* (A) and *RBSP3A* (B) mutants. A. Growth of stably transformed KRC/Y RCC cells with wild type and mutant *RASSF1A* (Cys65Arg and Val211Ala) without doxycycline (the gene is on) is presented in A. On day 6, the number of cells with wt *RASSF1A* was 3×10^5^ and the number of cells with mutant *RASSF1A* was 5×10^5^ (1.7 times more than wt). On day 10, the number of cells with wt *RASSF1A* was 6×10^5^ and the number of cells with mutant *RASSF1A* was 1.8×10^6^ (3 times more than wt). The effect of expression of wild type and mutant *RBSP3A* and *RBSP3B* on colony formation efficiency in KRC/Y cells is shown in B. Mutants were isolated from the N417 SCLC cell line (His139Tyr), the ovarian tumor biopsy T4 (three mutations: Asn31Asp, Pro79Ser and Glu87Lys) and the KRC/Y cell line (three mutations: Lys35Met, Asp103Gly and Leu181Pro). The number of blasticidin-resistant colonies compared to the empty pETE were: 90% for mutant from N417, 15% for mutant from T4 biopsy and 25% for mutant from KRC/Y. The number of colonies for wtRBSP3A were 5–10% compared to the pETE colonies.

We tested one *RASSF1A* gene, isolated from an RCC biopsy that contained two mutations (Cys65Arg and Val211Ala), for growth inhibition under cell culture conditions following transfection into the KRC/Y and prostate cancer LNCaP cells. In KRC/Y cells the mutated gene had a significantly reduced growth suppression activity (Figure 4A) while in LNCaP it had almost no suppressing activity (same as the empty vector, see [16], [17]).

In another experiment, we used *RBSP3* clones isolated from N417 SCLC cell line with a His139Tyr mutation. Again significant decrease in growth suppressor activity was observed (Figure 4B).Clearly, not all mutations found in this study would inactivate the *RBSP3* and *RASSF1* genes, and this may be especially true for mutants isolated from normal cells some of which could be polymorphisms. Indeed, different mutants of *RBSP3* had significantly different growth suppression activity (Figure 4B).

### Conclusions

By sequencing 327 *RASSF1A* and *RBSP3* clones, we detected 364 mutations with frequencies reaching 0.70 per 100 bp. Interestingly many clones contained more than 1 mutations (see Table S3A, B, C). Only one SNP was detected in *RASSF1A* ten clones (exon1α – AAG→CAG, K21Q) and it was excluded from the list of mutations [http://www.ncbi.nlm.nih.gov/SNP/snp_ref.cgi?locusId=11186]. No SNP were found in RBSP3 sequences.

The frequency of mutations was similar to other reported cases of somatic hypermutations found in Rho/TTF, MYC and BCL6 in large-cell lymphomas (MF was from 0.12 for MYC to 0.69 for BCL6). However it was significantly lower than for immunoglobulin genes (12.7 mutations per 100 bp, see [25]. However, for the first time we found high frequency of somatic mutations in different tissues including non-haematopoietic and in tumor suppressor genes contrary to the previous reports where oncogenes were studied.

As AccuPrime™*Pfx* DNA polymerase creates maximally one error in 3×10^6^ bp, our results proved that the observed hypermutation frequencies in the experiments could not be explained by erroneous performance of polymerases. In our experiment with SCID mice when AccuPrime™*Pfx* DNA polymerase and 25 cycles were used, 85% of *RBSP3* clones contained mutations.

During the growth of the same cell lines *in vitro*, 30% of *RBSP3* clones (also 25 cycles and AccuPrime™*Pfx* DNA polymerase) were mutant.

In experiments with *RASSF1A* (391 bp of the first and second exons), 65% of clones contained mutations (experiments with normal cells are not included). Moreover, in our experiments, we used different polymerases with different error rate (see M/M) and no significant differences in mutation frequency were observed, arguing against the generation of the mutations during PCR amplification. Different mutation frequencies between *in vivo* and *in vitro* experiments and in ESTs isolated from normal and cancer cells is an additional argument against the artificial nature of the hypermutation rate observed in *RBSP3* and *RASSF1* genes.

Mutations were detected with similar frequency both in cDNA and genomic DNA for *RBSP3* and *RASSF1*, however, no high mutability either on genomic or cDNA level were found for albumin, insulin, *GPR14*, TSG *p16/INK4a* or transcription elongation factor A (SII) *TCEA1*. Moreover, no mutations were found in experiments with SCID mice for 5 genes: *BLU*, *101F6*, *PL6*, mut*FUS1* and mut*P53*. Expression of *RBSP3* [10] in six tested samples differed almost 50-fold and on genomic level *RBSP3* was present usually in 3–8 copies [26]. Our previous experiments using marker NL3-001 located 90 kb apart from the *RASSF1A* demonstrated that in tumor cells this region in most cases is present in 1–5 copies [4], [5]. Still the frequency of mutations was almost the same. Thus number of template molecules didn't influence significantly mutability level. Moreover, repeated sequencing of the same plasmid and isolated by different persons and at different time gave identical results (6 *RASSF1A* and 6 *RBSP3* plasmid clones were sequenced) excluding frequent sequencing errors.

Both genes are CG rich however it seems that although high CG content can induce additional mutations it cannot explain the fact that two genes with significantly different CG content (*RASSF1A*, exons 1–2, 72.3%; *RASSF1A*, exons 1–6, 59.8% and *RBSP3*, exons 1–8, 54.3%) both possess high mutability while other genes with similar CG content (e.g. *GPR14*, 72.5%; *p16/INK4a*, 71.6%; insulin 61.6%) didn't show any high frequency of mutations.

Using the same PCR conditions plasmids containing *RBSP3* and *RASSF1A* were amplified from *E.coli* and no mutations were discovered arguing against generation of mutations during PCR amplification.

Experiments to find founder mutations with single-cell clones additionally confirm that mutations originate in the cell. Interestingly that from the single cell clone No. 9 we isolated plasmids with one, two or three mutations. This fact clearly showed how these mutations originate from one parental cell clone (Figure 2). Importantly after sequencing exons 3–5 of *RASSF1A* gene from KRC/Y we discovered founder mutation (destroying splice acceptor site) that was present in approximately 50% of 98 sequenced plasmid clones. This founder mutation appeared in all single cell clones and thus most likely it originated before we started this experiment.

For identification of tumor suppressor genes, we use the gene inactivation test, GIT [26], [27]. This test is based on the functional inactivation of the analyzed genes during tumor growth in SCID mice. Our hypothesis was that under selective pressure *in vivo* the introduced TSG must be inactivated in growing experimental (xenografted) tumors (by deletion, mutation, promoter methylation) as in the naturally growing tumors. The expression of the tested gene in the GIT was regulated by tetracycline and the level of expression was under physiological conditions. In our published papers [10], [23] wild type *RBSP3* and wild type and mutated *RASSF1A* genes were tested in GIT. The genes were PCR amplified from tumors and sequenced. In contrast to the wild type *RBSP3* and *RASSF1* genes, that were inactivated (i.e. deleted, non-expressed, mutated) in all 32 grown tumors, the mutant *RASSF1A* was not additionally mutated in any of four analyzed tumors. Importantly, in these GIT experiments we used direct sequencing of PCR products. These experiments showed that “founding mutations” really do exist.

Analysis of public EST databases confirmed our experimental data. It should be noted that the frequency of mutations in *RASSF1A* and *RBSP3* found in EST databases even using very stringent criteria was significantly higher than found in our experiments. MF for all mutations for *RBSP3* was 0.63 and for *RASSF1* it was 0.22. Probably, this discrepancy could account for the differences between the cell types analyzed in our experiments and in the EST database.

Unfortunately only 17 *RASSF1A* clones could be analysed because other EST sequences were either not sufficiently good or could be other isoforms of the *RASSF1* gene.

Interestingly, mutations of *RASSF1A* and *RBSP3* changing amino acids were found even in clones isolated from normal cell RNA, however, at a lower frequency than in cancer cells (MF ratios for cancer/normal sequences were 3.3 and 3, respectively). This difference for both genes was statistically significant (P<0.001) This probably reflects the selection for and the advantage of coding mutations during cancer progression. Important to mention that “normal” sequences include also non-annotated sequences so we cannot exclude that some of the “normal” sequences actually represent cancer cells.

In fact, these results correlate with the data from the mouse *in vivo* experiments that showed a higher frequency of mutations in SCID tumors than in the same cells grown *in vitro*. Interestingly, the same mutations were observed in cells grown *in vitro* and *in vivo*, in SCID mice (see Table S2B).

We have also experimentally tested whether *RASSF1A* (genomic DNA, exons1 and 2) harbored mutations in normal tissues and found one mutated clone out of 14 in normal kidney (normal control to T356, see section “Frequent mutations in *RASSF1A* in human carcinomas”). Important to note that so called “normal” kidney could be already partially transformed despite of normal phenotype because it was obtained from tissues adjacent to the tumor. We also sequenced complete *RASSF1A* cDNA from normal heart and detected six mutated clones out of 15 tested. All six heart mutated clones contained the same two mutations: L214L with codon changed from CTA to CTG and V236V with codon changed from GTA to GTG. Mutations in heart *RASSF1* cDNA were most likely SNP as they could be also found in other *RASSF1* clones in public databases (e.g. AC002481, NM_170713.2, NM_170714.1). In any case it is clear that mutations in *RASSF1* in normal cells are more rare than in cancer cells.

As we found mutations in all 5 coding exons of *RASSF1A* (the last 6^th^ exon contains only 48 amino acids = 144 bp). It means that other six known isoforms of *RASSF1* are also frequently mutated.

Exceptionally high level of germ line SNP mutations in *RASSF1A* found in several studies [8] support our data that the two genes we studied have rather frequent mutations even in normal cells.

The pattern of mutations was very different compared to those reported for AID and APOBEC enzymes and cannot be explained by polymerase errors. This is the first report of high mutation frequencies of *RASSF1* and *RBSP3* genes in different epithelial malignancies. In our preliminary paper [28] we analyzed mutations in *RASSF1A* gene in NPC samples and the results supported the present observations. In the NPC experiments 35 mutations were detected in 23 patients and mutations were considered real if at least two clones from the same patient contained the same mutation. Ten clones for each sample were sequenced in these experiments. Both DNA strands were sequenced.

At present, we don't know the nature of the mechanism responsible for this hypermutability, and only speculations could be done for its physiological function(s) in normal cells. There are several DNA polymerases in vertebrate cells that inaccurately copy templates and could be involved in generating hypermutations [29]. One of them, *POLH* (error rate 3×10^−2^), has a mutation target motif WA and may contribute to hypermutagenesis of immunoglobulin genes at A-T bases [30]. *POLH* is expressed in all tissues and, in principle, could cause hypermutations in non-haematopoietic cells. We found that 50% of all observed mutations in *RBSP3* happened in A or T surrounded by G or C. That means that the mutation target motif for 50% of mutations in *RBSP3* is SWS and is different from the *POLH* motif. It is reasonable to suggest that other(s) yet unknown DNA polymerase(s) may be responsible for the high mutability rates we report here and more than one polymerase contributes to hypermutations [29], [31].

Our results also argue that mutations are not completely random. They are not correlated with predicted numbers (Tables 1, 2, 3). For example according to statistical calculations our sequences of *RASSF1A* exons 1 and 2 should contain 0.026 nonsense mutations but in reality we detected 3 nonsense mutations, P<0.001 (see Table S1A). For *RASSF1A* exons 3–5 the predicted number of nonsense mutations is 0.037 and we found 3 such mutations, P<0.001 (see Table S1B). This fact may reflect the nature of cancers and normal tissues studied here. We cannot also exclude that these mutations still have some preferable motif(s).

We mentioned in the text that clonal selection for more aggressive growth of cancer cells could add to changing proportion of different mutations. In our previous paper [28] we also observed an unusual distribution of mutations. Among 35 detected mutations we found 30 transitions, 3 transversions, 2 deletions (frameshift), 3 nonsense (stop), 26 missense and only 4 were synonymous.

High frequency of mutations in different cancers and normal cells was reported earlier for P53 [32]. However, at present it is difficult to compare these results with our study as different methodologies were used and most likely different mechanisms of mutagenesis were involved.

When this manuscript was completed two new publications appeared in PNAS that support our observations and concept [33], [34].

Interestingly, in the paper of Yang et al. [35] hypermutability was demonstrated in damaged single-strand DNA formed at double–strand breaks in yeast *S. cerevisia*. Although yeast data may not apply to human cells, it is worthwhile to note that AP20 and LUCA regions where *RASSF1* and *RBSP3* are located were found extensively damaged (deletions, amplifications) in 90% of studied major epithelial cancers [2], [4], [5, see Introduction].

## Materials and Methods

### Ethics Statement

All work with mice was performed in special “Animal House” in MTC according to the standard rules. The study was done in accordance to the guidelines (incl. husbandry) issued by the STOCKHOLMS Norra Djurforsoksetiska Namnd (Animal Ethic Committee of North Stockholm).

Paired tumor/normal samples were obtained from the Blokhin Cancer Research Center, Russian Academy of Medical Sciences after surgical resection of primary tumors and stored in liquid nitrogen.. Top and bottom sections (3–5 µm thick) cut from frozen tumor tissues were examined histologically and only samples containing 70% or more tumor cells were used in the study. The samples were collected in accordance to the guidelines issued by the Ethic Committee of the Blokhin Cancer Research Center, Russian Academy of Medical Sciences (Moscow). All patients gave written informed consent that is available upon request. The study was done in accordance with the principles outlined in the Declaration of Helsinki.

### Cell lines and experiments with SCID mice

Cell lines were obtained from the MTC-KI (Stockholm, Sweden) cell lines collection. Cell and tumor growth assays were done as described previously [13], [16], [23], [26]. GIT was performed as described previous [16], [26], [27].

In brief, plasmid DNAs were purified using R.E.A.L. Prep kit (Qiagen, Valencia, CA). Transfections were performed using LipofectAMINE PLUS Reagent (Life Technologies, Rockville, MD) according to the manufacturer's protocol. After transfection, cells were selected with 5 µg/ml Blasticidin and 200 ng/ml doxycycline for two-four weeks. For colony formation assay cells were selected for 2 weeks, fixed, stained with Giemza and counted for transfection efficiency. For isolation of stably transfected cell clones, selection was done for four weeks. PCR positive clones from each recombinant were tested for expression using Northern hybridization and selected clones, 5×10^6^ cells/mouse, were inoculated subcutaneously with or without Matrigel (BD, Franklin Lakes, NJ) into six-week-old female SCID mice. Each mouse received only 1 injection. SCID mice were observed for tumor formation twice a week for up to seven weeks, if tumor formation was observed, tumors were measured using calipers. The tumors were explanted for DNA preparations.

### General methods

All molecular biology and microbiology procedures were performed as described previously [10], [13], [36]. DNA and RNA were isolated from total tumor samples containing less than 30% of non-tumor cells according to histopathology examination.

Construction of pETE vector and KRC/Y and LNCaP cell lines producing tetracycline trans-activator tTA were described in ref. [26].

### Polymerases used for PCR

In experiments with cell lines and biopsies we used natural Taq polymerase (New Englands Biolabs, Ipswich, MA, USA) and JumpStart™AccuTaqLA DNA Polymerase (Sigma-Aldrich, St. Louis, MO, USA). In some experiments (for comparison) we used AccuPrime™*Pfx* DNA polymerase (Invitrogen, Carlsbad, CA, USA). No significant difference was observed between these three polymerases. Usually 30 cycles were used.

In experiments with single cell clones and SCID mice AccuPrime™*Pfx* DNA polymerase and 25 cycles were employed.

Natural Taq polymerase has an error rate 4.5–5×10^−5^ (i.e. maximally 1 mistake per 200.000 bp) and the JumpStart™AccuTaqLA DNA Polymerase exhibits 6.5 fold higher fidelity. In many experiments, to exclude the possibility of generating mutations during the polymerization, we used the most error free polymerase available (15–26 fold better fidelity than ordinary Taq polymerase) AccuPrime™*Pfx* DNA polymerase and only 25 cycles.

The size of the *RBSP3B* is 1003 bp and the accuracy of ordinary Taq polymerase is approximately one error in 2×10^5^ bp. This means that after 30 cycles 15% of clones would be expected to contain mutation(s) in the *RBSP3* and after 25 cycles 12.5%. In the case of AccuPrime™*Pfx* DNA polymerase, after 30 cycles 1% of clones would be mutant and after 25 cycles only 0.84%. In our experiment with SCID mice, 85% of *RBSP3* clones contained mutations (AccuPrime™*Pfx* DNA polymerase, 25 cycles).

During the growth of the same cell lines *in vitro*, 30% of *RBSP3* clones (also 25 cycles and AccuPrime™*Pfx* DNA polymerase) were mutant.

### PCR and Sequencing

PCR primers were purchased from Invitrogen (Carlsbad, CA, USA). PCR was done as described earlier [2]. Initial denaturation was done for 2 min at 95°C following 25–30 cycles: 95°C for 15 sec, 64°C for 30 sec and extension at 68°C for 1 min per 1 kb.

*RBSP3A* and *RBSP3B*: gene fragments (ORF) have been obtained by PCR from cDNA isolated from different cell lines using the following primer sets, according to manufacturer's manual.

*RBSP3B*. 120C: 5′-GCGGCCGCCGCGCCGCGCACCCATGGACGGCCCGGCCATC-3′ (nucleotides 1-40) and HYA22C: 5′-AAAACAAAACAGGTAGGCATGGCCACATTC-3′ (nucleotides 1003-973). See GenBank Accession No. AJ575645

*RASSF1A*: genomic fragments (GenBank Accession No. AC002481).

Ex1–Ex2. F2A: 5′-GCCCAAAGCCAGCGAAGCAC-3′ (nucleotides 18051-18070) and EX2F2: 5′-ACCCAGGCAGCCCTCGAGAA-3′ (nucleotides 21066- 21047).

Ex3–Ex5. RassF1-2intrF: 5′-TGT CCA TGC TGG CCC ATC TTG C-3′ (nucleotides 26713-26734) and RassF1-5exR: 5′-CAC CTC CCC AGA GTC ATT TTC CTT C-3′ (nucleotides 27530-27554).

RASSF1A: cDNA fragment cDNA (ORF):

F2A: 5′-GCCCAAAGCCAGCGAAGCAC-3′ (nucleotides 97-116) and

F2B: 5′-AGCCATACCT GGCTACACCCACAGG-3′ (nucleotides 1343- 1319),

see GenBank Accession No. NM_007182

GPR14: genomic fragment (ORF).

GPR14F: 5′ - CCCATCTCAGGGAGTGTCCA - 3′ (nucleotides -52 - 33),

and GPR14R: 5′ - GTAGTTCCTGGTGAGCAGCGTGTAG - 3′ (nucleotides 966 - 942), see GenBank Accession No. NM_018949

TCEA1P2: genomic fragment (ORF).

TCEA1F: 5′ - TTTGTGAGGAAGGGGGCCTA - 3′ (nucleotides 705 - 724),

and TCEA1R: 5′ - ATATTTTGCCAATTCTTCCAACTCAACA - 3′ (nucleotides 1775 - 1748), see GenBank Accession No. X73534

pETE primers [26]:

LiTetF: 5′ - GCCTATATAAGCAGAGCTCGTTTAG - 3′

AtetR: 5′ - CCAAACTCATCAATGTATCTTATCA - 3′

Insulin: genomic fragment (ex1–ex3).

InsF: 5′-CTGTCACCCAGATCACTGTCCTTC-3′ (nucleotides 546-569) and InsR: 5′-GGGCTGCGTCTAGTTGCAGTAGTT-3′ (nucleotides 1702-1679), see GenBank Accession No. AY138590.1.

Albumin: cDNA fragment (ex12–ex15). AlbF: 5′-GAACCAGTTATGTGTGTTGCATGAGAA-3′ (nucleotides 1482 -1508), and AlbR: 5′-CCCACAGAAACTAGAAATCCTCTACCG-3′ (nucleotides 2181 -2155), see GenBank Accession No. NM_000477.3.

All experiments were performed using Gene Amp PCR System 9700 (Perkin Elmer, Foster City, CA, USA).

PCR products were cloned, using the TOPO TA cloning kit for sequencing (Invitrogen). Plasmid DNA was purified using the R.E.A.L.- Prep kit (Qiagen, Valencia, CA). Sequencing was done using an ABI 310 Sequencer (Applied Biosystems, Foster City, CA), according to the manufacturer's protocol.

### Bioinformatics

For *RASSF1A* only exons 1 and 2 with a total length of 357 bp (Acc.No. NM_007182) were analyzed. For *RBSP3* the longest isoform B (Acc.No. AJ575645; the total length is 831 bp) was analyzed. The gene sequences were searched against GenBank EST division, a collection of expressed sequence tags, or short, single-pass sequence reads from mRNA (cDNA). The statistically significant thresholds for the alignment (score) that provided elimination of alien mRNA sequences was set for *RASSF1A* at 462 and for *RBSP3* at 404. These thresholds were obtained from expertise estimation to cut off clusters of short and non-significant homologies to the query sequences. An additional manual refinement against low quality sequences was performed. Nucleotide similarity searches were performed with BLAST 2.2.

In all experiments we always compared a given sequence with the annotated sequences as shown in previous paragraph.

Probabilities of mutation frequency differences were calculated using Poisson distribution.

## Supporting Information

Table S1 (0.39 MB DOC)

Table S2 (0.16 MB DOC)

Table S3 (0.15 MB DOC)
