# Supplementary material for: High Mutability of the Tumor Suppressor Genes RASSF1 and RBSP3 (CTDSPL) in Cancer
Source: PLoS One. 2009 May 29;4(5):e5231. doi: 10.1371/journal.pone.0005231 (PMC2684631; doi:10.1371/journal.pone.0005231)
Supplement: Table S1 — (0.39 MB DOC) [file pone.0005231.s001.doc]

**Supporting information**

Table S1A. Mutations in *RASSF1A* (exons 1 and 2).

| No. | Codon position | Amino acid changes | Codon changes | tumors/cell lines |
| --- | --- | --- | --- | --- |
| 1 | -34 nt* |  | **G**→**A** | BL-2 *de novo* |
| 2 | -9 nt* |  | **A**→**G** | BL-2 |
| 3 | -9 nt* |  | **A**→**G** | BL-2 |
| 4 | -6 nt* |  | **C**→**G** | BL-2 |
| 5 | 2 | Ser to Thr | **T**CG→**A**CG | IARC171 |
| 6 | 3 | Gly to Glu | G**G**G→G**A**G | Ramos |
| 7 | 3 | Gly to Gly | GG**G**→GG**C** | T356(RCC) |
| 8 | 3 | Gly to Arg | **G**GG→**A**GG | KRC/Y |
| 9 | 4 | Glu to Gly | G**A**G→G**G**G | T356(RCC) cDNA |
| 10 | 6 | Glu to Asp | GA**G**→GA**C** | Caki1 |
| 11 | 7 | Leu to Pro | C**T**C→C**C**C | Ramos |
| 12 | 7 | Leu to Pro | C**T**C→C**C**C | BL-2 |
| 13 | 8 | Ile to Thr | A**T**T→A**C**T | TK10 |
| 14 | 8 | Ile to Thr | A**T**T→A**C**T | Mutu III |
| 15 | 10 | Leu to Pro | C**T**G→C**C**G | Ramos |
| 16 | 10 | Leu to Pro | C**T**G→C**C**G | T356 (RCC) |
| 17 | 12 | Glu to Gly | G**A**G→G**G**G | T356(RCC) cDNA |
| 18 | 13 | Leu to Pro | C**T**G→C**C**G | TK10 |
| 19 | 13 | Leu to Leu | CT**G**→CT**A** | TK10 |
| 20 | 15 | Pro to Leu | C**C**C→C**T**C | BL-2 |
| 21 | 16 | Ala to Ala | GC**T**→GC**C** | Mutu III |
| 22 | 17 | Gly to Arg | **G**GG→**A**GG | TK10 |
| 23 | 19 | Ala to Gly | G**C**T→G**G**T | Ramos |
| 24 | 20 | Gly to **del(G)** | **G**GG→-GG | Mutu III |
| 25 | 21 | Lys to Asn | AA**G**→AA**T** | BL-2 |
| 26 | 21 | Lys to Glu | **A**AG→**G**AG | BL-2 *de novo* |
| 27 | 21 | Lys to Glu | **A**AG→**G**AG | BL-2 *de novo* |
| 28 | 23 | Arg to His | C**G**C→C**A**C | BL-2 |
| 29 | 27 | Glu to Val | G**A**G→G**T**G | Mutu III |
| 30 | 28 | Arg to Arg | CG**T**→CG**G** | Ramos |
| 31 | 28 | Arg to Arg | CG**T**→CG**G** | BL-2 |
| 32 | 28 | Arg to Arg | CG**T**→CG**C** | BL-2 *de novo* |
| 33 | 29 | Ala to Ala | GC**C**→GC**A** | Ramos |
| 34 | 29 | Ala to Thr | **G**CC→**A**CC | IARC171 |
| 35 | 30 | Asn to Asp | **A**AC→**G**AC | IARC171 |
| 36 | 31 | Ala to Ser | **G**CG→**T**CG | BL-2 |
| 37 | 31 | Ala to Ser | **G**CG→**T**CG | Ramos |
| 38 | 32 | Leu to Leu | CT**G**→CT**A** | Ramos |
| 39 | 32 | Leu to Arg | C**T**G→C**G**G | BL-2 |
| 40 | 36 | Arg to Trp | **C**GG→**T**GG | IARC171 |
| 41 | 36 | Arg to Gln | C**G**G→C**A**G | TK10 |
| 42 | 36 | Arg to Gln | C**G**G→C**A**G | TK10 |
| 43 | 38 | Thr to Thr | AC**C**→AC**T** | TK10 |
| 44 | 39 | Ala to Val | G**C**G→G**T**G | TK164 |
| 45 | 39 | Ala to Val | G**C**G→G**T**G | IARC171 |
| 46 | 39 | Ala to Glu | G**C**G→G**A**G | Mutu III |
| 47 | 40 | Cys to Cys | TG**C**→TG**T** | IARC171 |
| 48 | 41 | Asn to Asp | **A**AC→**G**AC | Ramos |
| 49 | 41 | Asn to Asp | **A**AC→**G**AC | TK164 |
| 50 | 41 | Asn to **del(C)** | AA**C**→AA- | T356(RCC) |
| 51 | 41 | Asn to **del(C)** | AA**C**→AA- | T356(RCC) |
| 52 | 42 | Pro to Pro | CC**C**→CC**T** | BL-2 |
| 53 | 43 | Thr to Thr | AC**A**→AC**G** | BL-2 *de novo* |
| 54 | 43 | Thr to Thr | AC**A**→AC**G** | BL-2 *de novo* |
| 55 | 45 | Gln to Glu | **C**AG→**G**AG | IARC171 |
| 56 | 45 | Gln to **AMB** | **C**AG→**T**AG | Mutu III |
| 57 | 48 | Pro to Pro | CC**T**→CC**C** | BL-2 *de novo* |
| 58 | 50 | Arg to Cys | **C**GT→**T**GT | Mutu III |
| 59 | 50 | Arg to Arg | CG**T**→CG**C** | BL-2 *de novo* |
| 60 | 51 | Gly to Gly | GG**C**→GG**T** | TK10 |
| 61 | 52 | His to Tyr | **C**AC→**T**AC | BL-2 *de novo* |
| 62 | 53 | Arg to Arg | CG**C**→CG**A** | Mutu III |
| 63 | 53 | Arg to Arg | CG**C**→CG**A** | Ramos |
| 64 | 54 | Phe to Phe | TT**C**→TT**T** | Ramos |
| 65 | 54 | Phe to Ser | T**T**C→T**C**C | Mutu III |
| 66 | 54 | Phe to Leu | **T**TC→**C**TC | BL-2 |
| 67 | 56 | Pro to Ala | **C**CC→**G**CC | Ramos |
| 68 | 57 | Ala to Trp | **GC**G→**TG**G | BL-2 |
| 69 | 57 | Ala to Trp | **GC**G→**TG**G | BL-2 |
| 70 | 58 | Gly to Glu | G**G**G→G**A**G | TK10 |
| 71 | 59 | Pro to Leu | C**C**C→C**T**C | BL-2 |
| 72 | 60 | Ala to Val | G**C**C→G**T**C | Ramos |
| 73 | 61 | Thr to Thr | AC**G**→AC**T** | IARC171 |
| 74 | 62 | His to Arg | C**A**C→C**G**C | TK10 |
| 75 | 65 | Cys to Arg | **T**GC→**C**GC | Mutu III |
| 76 | 65 | Cys to Arg | **T**GC→**C**GC | Ramos |
| 77 | 66 | Asp to Val | G**A**C→G**T**C | BL-2 |
| 78 | 67 | Leu to Leu | CT**C**→CT**A** | Ramos |
| 79 | 68 | Cys to Cys | TG**T**→TG**C** | Ramos |
| 80 | 68 | Cys to Arg | **T**GT→**C**GT | KRC/Y |
| 81 | 70 | Asp to Ala | G**A**C→G**C**C | BL-2 |
| 82 | 70 | Asp to Tyr | **G**AC→**T**AC | TK164 |
| 83 | 70 | Asp to Gly | G**A**C→G**G**C | BL-2 *de novo* |
| 84 | 70 | Asp to Gly | G**A**C→G**G**C | BL-2 *de novo* |
| 85 | 71 | Phe to Ile | **T**TC→**A**TC | Ramos |
| 86 | 71 | Phe to Phe | TT**C**→TT**T** | Ramos |
| 87 | 73 | Trp to Cys | TG**G**→TG**T** | N356(RCC) |
| 88 | 73 | Trp to **del(G)** | T**G**G→T-G | T356(RCC) |
| 89 | 74 | Gly to Gly | GG**C**→GG**T** | BL-2 *de novo* |
| 90 | 75 | Val to Val | GT**C**→GT**T** | BL-2 |
| 91 | 76 | Val to Leu | **G**TG→**C**TG | Mutu III |
| 92 | 78 | Lys to Asn | AA**A**→AA**T** | BL-2 *de novo* |
| 93 | 78 | Lys to Lys | AA**A**→AA**G** | T356(RCC) cDNA |
| 94 | 81 | Gln to **AMB** | **C**AG→**T**AG | IARC171 |
| 95 | 82 | Cys to Ser | **T**GC→**A**GC | IARC171 |
| 96 | 83 | Ala to Ala | GC**G**→GC**A** | IARC171 |
| 97 | 83 | Ala to Val | G**C**G→G**T**G | BL-2 *de novo* |
| 98 | 84 | His to Arg | C**A**T→C**G**T | Ramos |
| 99 | 86 | Lys to Lys | AA**G**→AA**A** | T356(RCC) |
| 100 | 86 | Lys to **del(A)** | **A**AG→-AG | Ramos |
| 101 | 87 | Phe to Ile | **T**TC→**A**TC | T356 (RCC) |
| 102 | 90 | His to Arg | C**A**C→C**G**C | BL-2 |
| 103 | 91 | Tyr to Ser | T**A**C→T**C**C | Ramos |
| 104 | 92 | Arg to Ser | **C**GC→**A**GC | Ramos |
| 105 | 92 | Arg to Cys | **C**GC→**T**GC | Mutu III |
| 106 | 93 | Cys to Arg | **T**GC→**C**GC | Ramos |
| 107 | 94 | Arg to Arg | CG**C**→CG**A** | IARC171 |
| 108 | 96 | Leu to Leu | CT**C**→CT**T** | Ramos |
| 109 | 96 | Leu to Leu | CT**C**→CT**T** | Ramos |
| 110 | 96 | Leu to Pro | C**T**C→C**C**C | IARC171 |
| 111 | 96 | Leu to Pro | C**T**C→C**C**C | BL-2 |
| 112 | 97 | Val to Ala | G**T**C→G**C**C | IARC171 |
| 113 | 98 | Cys to Arg | **T**GC→**C**GC | T356(RCC) |
| 114 | 98 | Cys to **OPA** | TG**C**→TG**A** | Mutu III |
| 115 | 100 | Asp to Val | G**A**C→G**T**C | T356(RCC) |
| 116 | 101 | Cys to Cys | TG**T**→TG**C** | TK10 |
| 117 | 102 | Cys to Ser | **T**GC→**A**GC | BL-2 |
| 118 | 108 | Gly to Val | G**G**C→G**T**C | BL-2 |
| 119 | 110 | Glu to Lys | **G**AA→**A**AA | BL-2 |
| 120 | 110 | Glu to Gly | G**A**A→G**G**A | IARC171 |
| 121 | 110 | Glu to Glu | GA**A**→GA**G** | BL-2 *de novo* |
| 122 | 112 | Ala to Ala | GC**G**→GC**C** | Ramos |
| 123 | 114 | Glu to Lys | **G**AG→**A**AG | BL-2 |
| 124 | 114 | Glu to Glu | GA**G**→GA**A** | BL-2 *de novo* |
| 125 | 116 | Asp to Gly | G**A**C→G**G**C | T356(RCC) |
| 126 | 116 | Asp to Asn | **G**AC→**A**AC | TK10 |
| 127 | 117 | Thr to Ala | **A**CG→**G**CG | T356(RCC) |
| 128 | 118 | Asn to Ser | A**A**C→A**G**C | BL-2 |
| 129 | 119 | Val to Met | **G**TG→**A**TG | Mutu III |

* nucleotide positions.

Table S1B. Mutations in *RASSF1A* (exons 3,4 and 5)

In KRC/Y cell line.

| No. | Codon position | Amino acid changes | Codon changes | N clone |
| --- | --- | --- | --- | --- |
| 1 | 122 | Pro to Pro | CC**T**→CC**A** | N9 |
| 2 | 123 | Val to **del (T)** | G**T**G→G-G | N13 |
| 3 | 123 | Val to Gly | G**T**G→G**G**G | N3 |
| 4 | 124 | Glu to Gly | G**A**G→G**G**G | N3 |
| 5 | 124 | Glu to Val | G**A**G→G**T**G | N1 |
| 6 | 126 | Glu to Gly | G**A**G→G**G**G | N9 |
| 7 | 126 | Glu to Gly | G**A**G→G**G**G | N12 |
| 8 | 126 | Glu to Gly | G**A**G→G**G**G | N12 |
| 9 | 130 | Leu to Pro | C**T**T→C**C**T | N3 |
| 10 | 135 | Ile to **del(T)** | AT**T**→AT- | N3 |
| 11 | 135 | Ile to Val | **A**TT→**G**TT | N4 |
| 12 | 136 | Glu to **del(G)** | **G**AG→-AG | N3 |
| 13 | 137 | Gln to **AMB** | **C**AG→**T**AG | N6 |
| 14 | 138 | Lys to Arg | A**A**G→A**G**G | N12 |
| 15 | 139 | Ile to Asn | A**T**C→A**A**C | N9 |
| 16 | 139 | Ile to Asn | A**T**C→A**A**C | N9 |
| 17 | 139 | Ile to Asn | A**T**C→A**A**C | N9 |
| 18 | 139 | Ile to Thr | A**T**C→A**C**C | N4 |
| 19 | 139 | Ile to Thr | A**T**C→A**C**C | N4 |
| 20 | 139 | Ile to Thr | A**T**C→A**C**C | N4 |
| 21 | 139 | Ile to Thr | A**T**C→A**C**C | N4 |
| 22 | 139 | Ile to Thr | A**T**C→A**C**C | N4 |
| 23 | 140 | Lys to Arg | A**A**G→A**G**G | N2 |
| 24 | 140 | Lys to **AMB** | **A**AG→**T**AG | N10 |
| 25 | 143 | Asn to Asp | **A**AT→**G**AT | N3 |
| 26 | 144 | Ala to Val | G**C**C→G**T**C | N3 |
| 27 | 144 | Ala to Ala | GC**C**→GC**T** | N14 |
| 28 | 145 | Gln to His | CA**G**→CA**C** | N6 |
| 29 | 146 | Ile to Asn | A**T**C→A**A**C | N9 |
| 30 | 146 | Ile to Asn | A**T**C→A**A**C | N9 |
| 31 | 148 | Ser to Gly | **A**GC→**G**GC | N6 |
| 32 | 148 | Ser to Asn | A**G**C→A**A**C | N14 |
| 33 | 149 | Asn to Ser | A**A**C→A**G**C | N3 |
| 34 | 149 | Asn to Thr | A**A**C→A**C**C | N10 |
| 35 | 151 | Phe to Leu | **T**TC→**C**TC | N14 |
| 36 | 153 | Ser To Gly | **A**GC→**G**GC | N3 |
| 37 | 154 | Leu to Leu | **T**TG→**C**TG | N14 |
| 38 | 155 | Asn to Ser | A**A**C→A**G**C | N5 |
| 39 | 155 | Asn to Ser | A**A**C→A**G**C | N5 |
| 40 | 155 | Asn to Ser | A**A**C→A**G**C | N5 |
| 41 | 156 | Lys to Lys | AA**G**→AA**A** | N3 |
| 42 | 156 | Lys to Arg | A**A**G→A**G**G | N3 |
| 43 | 157 | Asp to Asn | **G**AC→**A**AC | N3 |
| 44 | 157 | Asp to Asn | **G**AC→**A**AC | N3 |
| 45 | 157 | Asp to Asn | **G**AC→**A**AC | N3 |
| 46 | 157 | Asp to Asn | **G**AC→**A**AC | N3 |
| 47 | 160 | Tyr to Cys | T**A**C→T**G**C | N11 |
| 48 | 162 | Gly to Asp | G**G**C→G**A**C | N11 |
| 49 | 163 | Phe to Tyr | T**T**C→T**A**C | N5 |
| 50 | 165 | Lys to **AMB** | **A**AG→**T**AG | N6 |
| 51 | 166 | Val to Ala | G**T**T→G**C**T | N9 |
| 52 | 168 | Leu to Pro | C**T**G→C**C**G | N9 |
| 53 | 169 | Lys to Glu | **A**AG→**G**AG | N14 |
| 54 | 169 | Lys to Arg | A**A**G→A**G**G | N14 |
| 55 | 170 | Leu to Met | **C**TG→**A**TG | N6 |
| 56 | 171 | Val to Ala | G**T**G→G**C**G | N14 |
| 57 | 171 | Val to Ala | G**T**G→G**C**G | N5 |
| 58 | 174 | Val to Val | GT**C**→GT**A** | N9 |
| 59 | 178 | Ser to Tyr | T**C**C→T**A**C | N14 |
| 60 | 180 | Lys to Arg | A**A**G→A**G**G | N3 |
| 61 | 189 | Arg to Arg | **C**GG→**A**GG | N6 |
| 62 | 193 | Gly to Glu | G**G**A→G**A**A | N3 |
| 63 | 196 | Thr to Thr | AC**A**→AC**T** | N1 |
| 64 | 196 | Thr to Ile | A**C**A→A**T**A | N11 |
| 65 | 196 | Thr to Thr | AC**A**→AC**T** | N1 |
| 66 | 196 | Thr to Thr | AC**A**→AC**T** | N1 |
| 67 | 197 | Ser to Arg | AG**T**→AG**G** | N2 |
| 68 | 197 | Ser to Ser | AG**T**→AG**C** | N3 |
| 69 | 200 | Arg to His | C**G**C→C**A**C | N3 |
| 70 | 202 | Thr to Ala | **A**CT→**G**CT | N4 |
| 71 | 206 | Leu to Leu | **C**TG→**T**TG | N12 |
| 72 | 208 | Lys to Arg | A**A**G→A**G**G | N5 |
| 73 | 210 | Ala to Thr | **G**CT→**A**CT | N4 |
| 74 | 210 | Ala to Ala | GC**T**→GC**C** | N14 |
| 75 | 210 | Ala to Ala | GC**T**→GC**C** | N14 |
| 76 | 212 | Lys to Arg | A**A**G→A**G**G | N4 |
| 77 | 212 | Lys to Glu | **A**AG→**G**AG | N3 |
| 78 | 214 | Leu to Pro | C**T**A→C**C**A | N3 |
| 79 | 216 | Val to Glu | G**T**G→G**A**G | N6 |
| 80 | 217 | Leu to Gln | C**T**G→C**A**G | N14 |
| 81 | 218 | Ser to Leu | T**C**A→T**T**A | N4 |
| 82 | 218 | Ser to Pro | **T**CA→**C**CA | N2 |
| 83 | 220 | Thr to Thr | AC**A**→AC**G** | N3 |
| 84 | 220 | Thr to Ala | **A**CA→**G**CA | N14 |
| 85 | 220 | Thr to **del(A)** | AC**A**→AC- | N2 |
| 86 | 221 | Arg to Gly | **A**GG→**G**GG | N3 |
| 87 | 221 | Arg to Gly | **A**GG→**G**GG | N4 |
| 88 | 223 | Arg to Arg | CG**T**→CG**C** | N13 |
| 89 | 224 | Glu to Val | G**A**A→G**T**A | N7 |
| 90 | 225 | Val to Ala | G**T**C→G**C**C | N3 |
| 91 | 225 | Val to Ala | G**T**C→G**C**C | N4 |
| 92 | 225 | Val to Ala | G**T**C→G**C**C | N4 |
| 93 | 225 | Val to Ala | G**T**C→G**C**C | N4 |
| 94 | 225 | Val to Ala | G**T**C→G**C**C | N4 |
| 95 | 225 | Val to Ala | G**T**C→G**C**C | N6 |
| 96 | 230 | Leu to Met | **C**TG→**A**TG | N11 |
| 97 | 230 | Leu to Pro | C**T**G→C**C**G | N3 |
| 98 | 232 | Lys to Arg | A**A**G→A**G**G | N8 |
| 99 | 232 | Lys to Arg | A**A**G→A**G**G | N8 |
| 100 | 232 | Lys to Arg | A**A**G→A**G**G | N8 |
| 101 | 233 | Phe to Ser | T**T**C→T**C**C | N14 |
| 102 | 236 | Val to Gly | G**T**A→G**G**A | N3 |
| 103 | 237 | Asp to Gly | G**A**T→G**G**T | N14 |
| 104 | 237 | Asp to Gly | G**A**T→G**G**T | N14 |
| 105 | 238 | Asp to Gly | G**A**C→G**G**C | N3 |
| 106 | 240 | Arg to Arg | CG**C**→CG**T** | N2 |
| 107 | 240 | Arg to Arg | CG**C**→CG**T** | N2 |
| 108 | 241 | Lys to Arg | A**A**G→A**G**G | N2 |
| 109 | 241 | Lys to Arg | A**A**G→A**G**G | N2 |
| 110 | 242 | Phe to Leu | **T**TT→**C**TT | N14 |
| 111 | 242 | Phe to Leu | **T**TT→**C**TT | N14 |
| 112 | 242 | Phe to Phe | TT**T**→TT**C** | N13 |
| 113 | 244 | Leu to Pro | C**T**C→C**C**C | N3 |
| 114 | 250 | Arg to Arg | CG**T**→CG**C** | N2 |
| 115 | 253 | Gln to Arg | C**A**A→C**G**A | N3 |
| 116 | 256 | Leu to Trp | T**T**G→T**G**G | N10 |
| 117 | 256 | Leu to Trp | T**T**G→T**G**G | N10 |
| 118 | 258 | Lys to Glu | **A**AG→**G**AG | N3 |
| 119 | 259 | Leu to Pro | C**T**G→C**C**G | N6 |
| 120 | 260 | Leu to Ser | T**T**G→T**C**G | N14 |
| 121 | 260 | Leu to Ser | T**T**G→T**C**G | N14 |
| 122 | 260 | Leu to Ser | T**T**G→T**C**G | N14 |
| 123 | 260 | Leu to Ser | T**T**G→T**C**G | N14 |
| 124 | 262 | Asp to Gly | G**A**T→G**G**T | N15 |
| 125 | 262 | Asp to Gly | G**A**T→G**G**T | N15 |
| 126 | 262 | Asp to Gly | G**A**T→G**G**T | N15 |
| 127 | 269 | Arg to Trp | **C**GG→**T**GG | N14 |
| 128 | 274 | Pro to Pro | CC**C**→CC**T** | N10 |
| 129 | 274 | Pro to Pro | CC**C**→CC**T** | N10 |
| 130 | 275 | Ser to Ser | AG**T**→AG**C** | N13 |
| 131 | 275 | Ser to Asn | A**G**T→A**A**T | N3 |
| 132 | 277 | Lys to Met | A**A**G→A**T**G | N3 |
| 133 | 277 | Lys to Gln | **A**AG→**C**AG | N3 |
| 134 | 279 | Leu to Met | **C**TG→**A**TG | N4 |
| 135 | 279 | Leu to Met | **C**TG→**A**TG | N4 |
| 136 | 280 | Ser to Gly | **A**GC→**G**GC | N2 |
| 137 | 282 | Val to Ala | G**T**C→G**C**C | N1 |
| 138 | 283 | Leu to Pro | C**T**G→C**C**G | N3 |
| 139 | 283 | Leu to Leu | **C**TG→**T**TG | N4 |
| 140 | 285 | Glu to Gly | G**A**A→G**G**A | N3 |
| 141 | 287 | Asp to Ala | G**A**C→G**C**C | N3 |
| 142 | 286 | Asn to **del(A,T)** | A**AT**→A-- | N3 |
| 143 | 290 | Glu to Glu | GA**G**→GA**A** | N13 |
| 144 | 291 | Val to Met | **G**TG→**A**TG | N6 |
| 145 | 291 | Val to Ile | **G**TC→**A**TC | N13 |
| 146 | 26735 | Border’s mutation | **A**→**G** | all |
