# Supplementary material for: High Mutability of the Tumor Suppressor Genes RASSF1 and RBSP3 (CTDSPL) in Cancer
Source: PLoS One. 2009 May 29;4(5):e5231. doi: 10.1371/journal.pone.0005231 (PMC2684631; doi:10.1371/journal.pone.0005231)
Supplement: Table S2 — (0.16 MB DOC) [file pone.0005231.s002.doc]

**Supporting information**

Table S2A. Mutations in *RBSP3* (cDNA, splicing forms A and B) in human cancer, biopsies and cell lines.

| No. | Codon position | | Amino acid changes | Codon changes | Tumors/cell lines |
| --- | --- | --- | --- | --- | --- |
|  | A form | B form |  |  |  |
| 1 |  | 1 | Met to **del(A)** | **A**TG→-TG | N417^¤^ |
| 2 |  | 7 | Ile to Leu | **A**TC→**C**TC | RCC^#^ |
| 3 | 11 |  | Thr to Ala | **A**CC→**G**CC | OC^*^ |
| 4 |  | 13 | Pro to Ser | **C**CC→**T**CC | RCC^+^ |
| 5 |  | 17 | Glu to Glu | GA**G**→GA**A** | BC |
| 6 | 31 |  | Asn to Asp | **A**AC→**G**AC | OC |
| 7 | 69 |  | Leu to Pro | C**T**G→C**C**G | OC |
| 8 |  | 72 | Glu to Gly | G**A**G→G**G**G | RCC |
| 9 |  | 78 | Lys to Arg | A**A**G→A**G**G | RCC |
| 10 | 79 |  | Pro to Ser | **C**CA→**T**CA | OC |
| 11 | 87 |  | Glu to Lys | **G**AG→**A**AG | OC |
| 12 |  | 103 | Asp to Gly | G**A**C→G**G**C | OC |
| 13 |  | 103 | Asp to Gly | G**A**C→G**G**C | OC |
| 14 |  | 121 | Ser to Pro | **T**CG→**C**CG | BC |
| 15 |  | 127 | Asn to Ser | A**A**T→A**G**T | RCC |
| 16 |  | 132 | Val to Gly | G**T**T→G**G**T | BC |
| 17 |  | 132 | Val to Gly | G**T**T→G**G**T | BC |
| 18 |  | 132 | Val to Gly | G**T**T→G**G**T | BC |
| 19 |  | 132 | Val to Gly | G**T**T→G**G**T | BC |
| 20 | 138 |  | Pro to Ser | **C**CA→**T**CA | N417 |
| 21 | 139 |  | His to Tyr | **C**AT→**T**AT | N417 |
| 22 |  | 160 | Leu to Ile | **C**TT→**A**TT | OC |
| 23 | 150 |  | Leu to Pro | C**T**T→C**C**T | N417 |
| 24 | 164 |  | Ala to Val | G**C**A→G**T**A | RCC |
| 25 | 169 |  | Asp to Gly | G**A**C→G**G**C | OC |
| 26 |  | 192 | Leu to Pro | C**T**C→C**C**C | RCC |
| 27 |  | 198 | Val to Ala | G**T**T→G**C**T | RCC |
| 28 |  | 219 | Ile to Val | **A**TC→**G**TC | RCC |
| 29 |  | 220 | Ile to Val | **A**TT→**G**TT | BC |
| 30 |  | 222 | Asp to Gly | G**A**C→G**G**C | RCC |
| 31 |  | 223 | Asn to Ser | A**A**T→A**G**T | BC |
| 32 |  | 245 | Met to Ile | AT**G**→AT**T** | RCC |
| 33 |  | 261 | Ser to Gly | **A**GC→**G**GC | OC |
| 34 |  | 264 | Asp to Gly | G**A**C→G**G**C | RCC |
| 35 |  | 265 | Asp to Gly | G**A**C→G**G**C | BC |
| 36 |  | 266 | Val to Ala | G**T**G→G**C**G | RCC |
| 37 |  | 267 | Tyr to His | **T**AC→**C**AC | RCC |
| 38 |  | 274 | Cys to Arg | **T**GC→**C**GC | BC |

^¤^ N417 - small cell lung carcinoma cell line

^#^ RCC - renal cancer carcinoma biopsy

*OC - ovarian carcinoma biopsy

^+^ BC - breast carcinoma biopsy

Table S2B. Mutations in *RBSP3* (splicing forms A and B) in SCID experiments.

| No. | Codon position | | Amino acid changes | Codon changes | Cel lines |
| --- | --- | --- | --- | --- | --- |
|  | A form | B form |  |  |  |
| 1 | 1 |  | Met to Lys | A**T**G→A**A**G | ACC-LC5^#^, *in vivo* |
| 2 | 2 |  | Asp to His | **G**AC→**C**AC | KRC/Y, *in vivo* |
| 3 | 5 |  | Ala to **del(G)** | **G**CC→**-**CC | KRC/Y, *in vivo* |
| 4 | 5 |  | Ala to Pro | **G**CC→**C**CC | KRC/Y, *in vivo* |
| 5 | 7 |  | Ile to Leu | **A**TC→**C**TC | KRC/Y, *in vivo* |
| 6 |  | 21 | Pro to Leu | C**C**G→C**T**G | KRC/Y, *in vivo* |
| 7 | 28 |  | Ser to Pro | **T**CC→**C**CC | KRC/Y, *in vivo* |
| 8 |  | 30 | Cys to Arg | **T**GC→**C**GC | ACC-LC5, *in vivo* |
| 9 | 43 |  | Leu to **del(T)** | CT**T**→CT- | ACC-LC5, *in vivo* |
| 10 | 44 |  | Ser to Gly | **A**GC→**G**GC | KRC/Y, *in vitro* |
| 11 |  | 47 | Phe to Ser | T**T**C→T**C**C | KRC/Y, *in vivo* |
| 12 |  | 49 | Cys to Arg | **T**GC→**C**GC | KRC/Y, *in vivo* |
| 13 |  | 77 | Gln to His | CA**G**→CA**C** | ACC-LC5, *in vivo* |
| 14 |  | 77 | Gln to Arg | C**A**G→C**G**G | ACC-LC5, *in vivo* |
| 15 |  | 78 | Lys to Arg | A**A**G→A**G**G | KRC/Y, *in vitro* |
| 16 |  | 88 | Pro to Leu | C**C**A→C**T**A | KRC/Y, *in vitro* |
| 17 |  | 103 | Asp to Gly | G**A**C→G**G**C | KRC/Y, *in vitro* |
| 18 |  | 105 | Gly to Gly | GG**A**→GG**G** | KRC/Y, *in vivo* |
| 19 | 111 |  | Phe to Leu | **T**TT→**C**TT | KRC/Y, *in vivo* |
| 20 |  | 148 | Arg to Arg | CG**G**→CG**A** | KRC/Y, *in vivo* |
| 21 | 142 |  | Glu to Gly | G**A**G→G**G**G | ACC-LC5, *in vivo* |
| 22 | 142 |  | Glu to Gln | **G**AG→**C**AG | KRC/Y, *in vivo* |
| 23 | 145 |  | Gln to **del(C)** | **C**AG→**-**AG | KRC/Y, *in vivo* |
| 24 |  | 157 | Arg to Gly | **A**GG→**G**GG | ACC-LC5, *in vivo* |
| 25 |  | 162 | Phe to Leu | **T**TT→**C**TT | KRC/Y, *in vitro* |
| 26 |  | 166 | Leu to Pro | C**T**C→C**C**C | KRC/Y, *in vivo* |
| 27 |  | 167 | Phe to Leu | **T**TT→**C**TT | ACC-LC5, *in vivo* |
| 28 |  | 175 | Ala to Ala | GC**A**→GC**C** | KRC/Y, *in vivo* |
| 29 |  | 175 | Ala to Ala | GC**A**→GC**T** | KRC/Y, *in vivo* |
| 30 |  | 181 | Leu to Pro | C**T**C→C**C**C | ACC-LC5, *in vivo* |
| 31 |  | 192 | Leu to Pro | C**T**C→C**C**C | KRC/Y, *in vivo* |
| 32 |  | 192 | Leu to Pro | C**T**C→C**C**C | KRC/Y, *in vitro* |
| 33 |  | 192 | Leu to Leu | CT**C**→CT**T** | ACC-LC5, *in vivo* |
| 34 | 186 |  | Cys to Arg | **T**GT→**C**GT | ACC-LC5, *in vivo* |
| 35 | 207 |  | Val to Ala | G**T**G→G**C**G | KRC/Y, *in vitro* |
| 36 |  | 219 | Ile to Thr | A**T**C→A**C**C | KRC/Y, *in vitro* |
| 37 | 210 |  | Val to Val | GT**T**→GT**C** | KRC/Y, *in vitro* |
| 38 |  | 223 | Asn to Asp | **A**AT→**G**AT | ACC-LC5, *in vivo* |
| 39 | 214 |  | Pro to **del(C)** | **C**CT→-CT | KRC/Y, *in vivo* |
| 40 |  | 229 | Ile to Val | **A**TC→**G**TC | KRC/Y, *in vivo* |
| 41 | 238 |  | Glu to Gly | G**A**G→G**G**G | KRC/Y, *in vivo* |
| 42 | 251 |  | Arg to Gln | C**G**G→C**A**G | ACC-LC5, *in vivo* |
| 43 |  | 270 | Leu to Leu | CT**G**→CT**A** | KRC/Y, *in vivo* |
| 44 |  | 272 | Arg to Arg | AG**A**→AG**G** | KRC/Y, *in vivo* |
| 45 | 831bp* |  |  | **T**→**C** | ACC-LC5, *in vivo* |
| 46 |  | 902bp* |  | **T**→**C** | KRC/Y, *in vivo* |
| 47 |  | 909bp* |  | **A**→**G** | KRC/Y, *in vivo* |
| 48 |  | 916bp* |  | **T→C** | KRC/Y, *in vivo* |
| 49 |  | 921bp* |  | **T**→**C** | KRC/Y, *in vitro* |
| 50 |  | 921bp* |  | **T**→**C** | KRC/Y, *in vivo* |
| 51 |  | 930bp* |  | **T**→**C** | ACC-LC5, *in vivo* |

* noncoding region

^#^ACC-LC5 - small cell lung carcinoma cell line
