# Supplementary material for: High Mutability of the Tumor Suppressor Genes RASSF1 and RBSP3 (CTDSPL) in Cancer
Source: PLoS One. 2009 May 29;4(5):e5231. doi: 10.1371/journal.pone.0005231 (PMC2684631; doi:10.1371/journal.pone.0005231)
Supplement: Table S3 — (0.15 MB DOC) [file pone.0005231.s003.doc]

**Supporting information**

Table S3A. Clones with more than 1 mutations in RASSF1 (ex1-ex2).

|  | Cell lines/Tumor | Number of mutations | Mutations |
| --- | --- | --- | --- |
| 1 | IARC171 | 2 | N30D (AAA→GAC), R36W (CGG→TGG) |
| 2 |  |  | A39V (GCG→GTG), V197A (GCT→GCC) |
| 3 |  |  | A29T (GCC→ACC), Q81Stop (CAG→TAG) |
| 4 |  |  | C82S (TGC→AGC), L96P (CTC→CCC) |
| 5 | BL2 | 4 | L32R (CTG→CGG), D66V (GAC→GTC), D70A (GAC→GCC), L96P (CTC→CCC) |
| 6 |  |  | L7P (CTC→CCC), G108V (GGC→GTC), E110K (GAA→AAA), E114K (GAG→AAG) |
| 7 |  | 3 | K21N (AAG→AAT), C102S (TGC→AGC), N118S (AAC→AGC) |
| 8 |  |  | P42P (CCC→CCT), V75V (GTC→GTT), K78N (AAA→AAT) |
| 9 |  | 2 | P15L (CCC→CTC), H90R (CAC→CGC) |
| 10 |  |  | R23H (CGC→CAC), P59L (CCC→CTC) |
| 11 |  |  | R28R (CGT→CGG, A57W (GCG→TGG) |
| 12 | BL2 de novo | 2 | T43T (ACA→ACG), E110E (GAA→GAG) |
| 13 |  |  | F54L (TTC→CTC), E114E (GAG→GAA) |
| 14 |  |  | D70G (GAC→GGC), G74G (GGC→GGT) |
| 15 | RAMOS | 7 | A19G (GCT→GGT), N41D (AAC→GAC), R53R (CGC→CGA), A60V (GCC→GTC), L67L (CTC→CTA), C68C(TGT→TGC), A112A(GCG→GCC) |
| 16 |  | 4 | L7P (CTC→CCC), P56A (CCC→GCC), R92S (CGC→AGC), L96L (CTC→CTT) |
| 17 |  | 3 | G3E (GGG→GAG), F54F (TTC→TTT), C65R (TGC→CGC) |
| 18 |  |  | L10P (CTG→CCG), F71I (TTC→ATC), C93R (TGC→CGC) |
| 19 |  |  | R28R (CGT→CGG), A31S (GCG→TCG), L96L (CTC→CTT) |
| 20 | mutuIII | 3 | A16A (GCT→GCC), G20del (GGG→GG-), R50C (CGT→TGT) |
| 21 |  |  | A39E (GCG→GAG), Q45Stop (CAG→TAG), V76L (GTG→CTG) |
| 22 |  | 2 | I8T (ATT→ACT), F54S (TTC→TCC) |
| 23 |  |  | E27V (GAG→GTG), V119M (GTG→ATG) |
| 24 |  |  | R53R (CGC→CGA), C65R (TGC→CGC) |
| 25 | TK10 | 4 | I8T (ATT→ACT), K21Q (AAG→CAG), C101C (TGT→TGC), D116N (GAC→AAC) |
| 26 | TK164 | 2 | N41D (AAC→GAC), D70Y (GAC→TAC) |
| 27 | T356 (RCC) | 3 | N41del (AAC→AA-), C98R (TGC→CGC), T117A (ACG→GCG) |
| 28 |  |  | N41del (AAC→AA-), K86K (AAG→AAA), D116G (GAC→GGC) |
| 29 |  | 2 | G3G (GGG→GGC), W73del (TGG→TG-) |

Table S3B. Clones with more than 1 mutations in RASSF1 (ex3-ex5).

|  | KRC/Y  Cell clones | Number of mutations | Mutations |
| --- | --- | --- | --- |
| 1 | N3 | 4 | N149S (AAC→AGC), V225A (GTC→GCC), V236G (GTA→GGA), D238G (GAC→GGC) |
| 2 | N3 |  | R200H (CGC→CAC), L214P (CTA→CCA), K258E (AAG→GAG), N286del (AAT→A--) |
| 3 | N4 |  | T202A (ACT→GCT), A210T (GCT→ACT), R221G (AGG→GGG), L279M (CTG→ATG) |
| 4 | N4 |  | K212R (AAG→AGG), S218L (TCA→TTA), K232K (AAG→AGG), L283L (CTG→TTG) |
| 5 | N10 |  | K140Stop (AAG→TAG), A210A (GCT→GCC), L256W (TTG→TGG), P274P (CCC→CCT) |
| 6 | N10 |  | N149T (AAC→ACC), A210A (GCT→GCC), L256W (TTG→TGG), P274P (CCC→CCT) |
| 7 | N16 |  | F242F (TTT→TTC), S275S (AGT→AGC), E290E (GAG→GAA), V291M (GTG→ATG) |
| 8 | N3 | 3 | S153G (AGC→GGC), S197S (AGT→AGC), S275N (AGT→AAT) |
| 9 | N3 |  | N143D(AAT→GAT), K156K (AAG→AAA), L244P (CTC→CCC) |
| 10 | N3 |  | E124G(GAG→GGG), A144V (GCC→GTC), T220T(ACA→ACG) |
| 11 | N6 |  | S148G(AGC→GGC),R189R(CGG→AGG),V291M (GTG→ATG) |
| 12 | N6 |  | Q137Stop (CAG→TAG), L170M (CTG→ATG), L259P (CTC→CTG) |
| 13 | N8 |  | T196I (ACA→ATA), L230M (CTG→ATG), K232R (AAG→AGG) |
| 14 | N9 |  | E126G (GAG→GGG), I139N (ATC→AAC), I146N (ATC→AAC) |
| 15 | N9 |  | P122P (CCT→CCA), I139N (ATC→AAC), I146N (ATC→AAC) |
| 16 | N5 |  | N155S (AAC→AGC), F163Y (TTC→TAC), F242L (TTT→CTT) |
| 17 | N5 |  | N155S (AAC→AGC), V171A (GTG→GCG), F242L (TTT→CTT) |
| 18 | N21 |  | Q145H(CAG→CAC),V216E (GTG→GAG),V225A (GTC→GCC) |
| 19 | N9 |  | I139N (ATC→AAC),V166A (GTT→GCT),V174V (GTC→GTA) |
| 20 | N14 |  | F151L (TTC→CTC),K169E (AAG→GAG), S178Y (TCC→TAC) |
| 21 | N14 |  | L154L (TTG→CTG),S148N (AGC→AAC),D237G (GAT→GGT) |
| 22 | N3 | 2 | K212E (AAG→GAG), L230P (CTG→CCG) |
| 23 | N14 |  | T220A (ACA→GCA),D237G (GAT→GGT) |
| 24 | N1 |  | E124V (GAG→GTG), V282A (GTC→GCC) |
| 25 | N2 |  | R240R (CGC→CGT), K241R (AAG→AGG) |
| 26 | N3 |  | I135del (ATT→AT-), E136del (GAG→-AG) |
| 27 | N3 |  | V123G (GTG→GGG), R221G (AGG→GGG) |
| 28 | N4 |  | I135V (ATT→GTT), L279M (CTG→ATG) |
| 29 | N2 |  | R240R (CGC→CGT), K241R (AAG→AGG) |
| 30 | N11 |  | Y160C (TAC→TGC), G162D (GGC→GAC) |
| 31 | N4 |  | I139T (ATC→ACC), V225A (GTC→GCC) |
| 32 | N3 |  | V123del (GTG→G-G), R223R (CGT→CGC) |
| 33 | N14 |  | A144A (GCC→GCT), K169R (AAG→AGG) |
| 34 | N12 |  | E126G (GAG→GGG), L168P (CTG→CCG) |
| 35 | N22 |  | R250R (CGT→CGC), S280G (AGC→GGC) |
| 36 | N29 |  | K138R (AAG→AGG), L206L (CTG→TTG) |
| 37 | N4 |  | I139T (ATC→ACC), V225A (GTC→GCC) |
| 38 | N4 |  | I139T (ATC→ACC), V225A (GTC→GCC) |
| 39 | N5 |  | N155S (AAC→AGC), K208R (AAG→AGG) |
| 40 | N4 |  | I139T (ATC→ACC), V225A (GTC→GCC) |
| 41 | N14 |  | V171A (GTG→GCG), L217Q (CTG→CAG) |
| 42 | N3 |  | L130P (CTT→CCT), G193E (GGA→GAA) |
| 43 | N2 |  | K140R (AAG→AGG),S197R (AGT→AGG) |
| 44 | N3 |  | K156R (AAG→AGG), K180R (AAG→AGG) |

Table S3C. Clones with more than 1 mutations in RBSP3.

|  | Cell lines/Tumor | Number of mutations | Mutations |
| --- | --- | --- | --- |
| 1 | RCC | 3 | P13S (CCC→TCC), E72G (GAG→GGG), I219V (ATC→GTC) |
| 2 |  |  | I7L (ATC→CTC), K78R (AAG→AGG), V198A (GTT→GCT) |
| 3 |  | 2 | M245I (ATG→ATT), Y267H (TAC→CAC) |
| 4 |  |  | D222G (GAC→GGC), V266A (GTG→GCG) |
| 5 | ovary | 3 | N31D (AAC→GAC), P79S (CCA→TCA), E87K (GAG→AAG) |
| 6 |  | 2 | T11A (ACC→GCC), D169G (GAC→GGC) |
| 7 |  |  | L160I (CTT→ATT), S261G (AGC→GGC) |
| 8 | breast | 4 | E17E (GAG →GAA), S121P (TCG→CCG), I220V (ATT→GTT), N223S (AAT→AGT) |
| 9 |  | 2 | V132G (GTT→GGT), D265G (GAC→GGC) |
| 10 |  |  | V132G (GTT→GGT), C274R (TGC→CGC) |
|  | SCID experiment |  |  |
| 11 | *in vivo* | 3 | L43del (CTT→CT-),E142G (GAG→GGG), R251Q (CGG→CAG) |
| 12 |  |  | M1K (ATG→AAG), C186R (TGT→CGT), 831(T→C) |
| 13 |  | 2 | S28P (TCC→CCC), E238G (GAG→GGG) |
| 14 |  |  | C30R (TGC→CGC), Q77R (CAG→CGG) |
| 15 |  |  | P21L (CCG→CTG), I229V (ATC→GTC) |
| 16 |  |  | A5del (GCC→-CC), Q77H (CAG→CAC) |
| 17 |  |  | R157G (AGG→GGG), 930 (T→C) |
| 18 | *in vitro* | 2 | K78R(AAG→AGG), F162L (TTT→CTT) |
| 19 |  |  | P88L (CCA→CTA), L192P (CTC→CCC) |
| 20 |  |  | S44G(AGC→GGC), V210V (GTT→GTC), |
